# Bronchial Carcinoid Tumour as a Rare Cause of Cushing’s Syndrome in Children: A Case Report and Review of Literature

**DOI:** 10.4274/jcrpe.galenos.2019.2019.0156

**Published:** 2020-11-25

**Authors:** Rahul Saxena, Manish Pathak, Ravindra Shukla, Arvind Sinha, Poonam Elhence, Jyotsna N. Bharti, Pushpinder Khera

**Affiliations:** 1 All India Institute of Medical Sciences, Department of Pediatric Surgery, Jodhpur, India; 2 All India Institute of Medical Sciences, Department of Endocrinology, Jodhpur, India; 3 All India Institute of Medical Sciences, Department of Pathology, Jodhpur, India; 4 All India Institute of Medical Sciences, Department of Diagnostic and Interventional Radiology, Jodhpur, India

**Keywords:** Paediatric Cushing’s syndrome, Ectopic ACTH syndrome, paediatric bronchial carcinoid

## Abstract

Cushing’s syndrome (CS) is rare in childhood and adolescence. The most common paediatric cause of CS is exogenous administration of glucocorticoids; either topical, inhaled or oral corticosteroids. Endogenous causes can be classified into adrenocorticotropic hormone (ACTH) independent and ACTH dependent causes. Herein, we report our experience of managing a 12 year old girl who presented with features of CS and was found to have an ectopic, ACTH-secreting bronchial carcinoid tumour, which was resected surgically. Our patient was managed successfully by multidisciplinary approach and has recovered from hypertension and Cushing’s habitus. The English language literature was searched from 2019 back, using PubMed, Google and Google Scholar. Keywords used for the search were; “Ectopic ACTH syndrome (EAS) in children”, “bronchial carcinoid in children” and “Cushing’s Syndrome in children”. Children with bronchial carcinoid tumours causing EAS were identified. Case variables such as age, sex, type of carcinoid, investigations, surgery, recurrences and outcome were reviewed. Fourteen cases of paediatric bronchial carcinoid producing ACTH were found with a mean age of 15.8 years and female preponderance. Most of the patients had a right lung lesion and histological appearance was typical of carcinoid tumour. Bronchial carcinoid is extremely rare in children and only 4% are associated with CS. The postoperative treatment of CS is challenging with a high prevalence of hypertension, increased body mass index and visceral fat mass, impaired cognitive function and decreased quality of life. A careful follow up is indispensable for monitoring recurrence of carcinoid and complete remission of CS.

## Introduction

Paediatric Cushing’s syndrome (CS), is a condition which occurs due to excessive amount of glucocorticoids in body, either produced endogenously or administered exogenously. The most common cause for the condition is iatrogenic, like in adults due to excessive administration of glucocorticoids. The endogenous paediatric CS is a rare condition, which is broadly classified into adrenocorticotropic hormone (ACTH) dependent and ACTH independent CS. When the excessive ACTH is secreted by pituitary adenoma, it is called as CS and if the source of ACTH production is outside pituitary, it is called as, Ectopic ACTH syndrome (EAS). Athough rare, the bronchial carcinoids are the most common causes for EAS in children. The overall incidence for bronchial carcinoids is 3-5 tumors per million people per year and 4% of pulmonary carcinoids are associated with CS. The median age of presentation is 9.5 years, with a female predominance. We describe a case of ectopic ACTH secreting bronchial carcinoid presented to us with symptoms and signs of CS and review the present literature for paediatric cases of EAS due to bronchial carcinoid.

Literature review was performed from the year 2019 back to the oldest available report in English, to analyse all cases of bronchial carcinoid tumours causing EAS in children. The online databases searched were PubMed/MEDLINE, Google Scholar and Google using the following keywords: “EAS in children”; “Bronchial carcinoid in children”; and “Cushing’s Syndrome  in children”. All articles that described paediatric patients with bronchial carcinoid tumours causing EAS were identified. Case variables such as age, sex, type of carcinoid, diagnosis, surgery and recurrences were reviewed. We also describe a teenage girl with an ectopic ACTH-secreting bronchial carcinoid tumour who presented with symptoms and signs of CS.

## Literature Search

Fourteen paediatric and adolescent patients with bronchial carcinoid tumours causing EAS were identified from nine case series and reports (1,2,3,4,5,6,7,8,9) excluding our patient. The mean age of these patients was 15.8±3.36 years. There were nine females (70%) and four male (30%) children and in one case gender of the patient was not specified. These cases included one atypical carcinoid tumour and four typical carcinoid tumours, mostly involving right lung. There was lymph node metastasis in six patients. All of them were managed by surgical excision of the tumour, although one patient underwent bilateral adrenalectomy due to relapse, two patients had bilateral adrenalectomy and one patient underwent hypophysectomy prior to surgery. There was one death reported and three patients had recurrence.

In a major series of ninety patients with EAS reported by Ilias et al (10), 35 patients with bronchial carcinoid tumours causing EAS were included but the ages ranged from 8-72 years (Table 1) with almost half of the patients having lymph node involvement requiring lymph node dissection. There were three deaths and two relapses reported.

**Figure 1:**
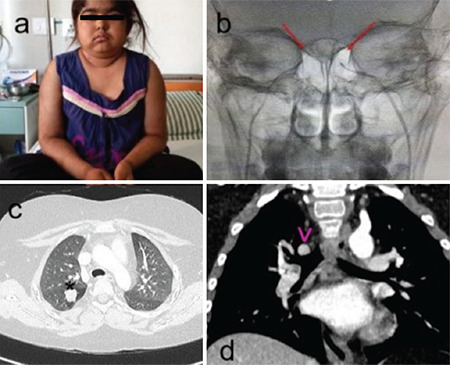
(a) Pre-operative photograph of patient showing cushingoid facies, hirsutism and obesity, (b) bilateral inferior petrosal sinus sampling with red arrows indicating the microcatheters in petrosal sinuses, (c) axial and, (d) coronal view of computed tomography chest showing a smoothly margined nodule of 15 mm diameter in the apical segment of the right upper lobe

A 12-year old girl presented with complaints of excessive weight gain, dry skin and weakness of limbs. There was no history of steroid intake or previous illness. On examination, she showed typical Cushing’s habitus, including “moon face”, abdominal striae, growth retardation, muscle weakness, dry and thick skin and excessive hair growth all over her body (Figure 1a).

She was hypertensive (130/90 mmHg) with a body mass index (BMI) of 22.7 kg/cm^2^ (height 134.5 cm and weight of 41 kg) with BMI standard deviation score (SDS) of 1.22. Her SDS for height for age and weight for age were -2.2, and -0.10 respectively. On evaluation, her midnight and evening serum cortisols concentrations were high (Table 2). A low dose dexamethasone suppression test was done to confirm CS which again showed non-suppressed serum cortisol (26.94 µg/dL). A high dose dexamethasone suppression test (HDDST) showed <50% suppression (baseline serum cortisol=59.55 µg/dL; suppressed cortisol=35.67 µg/dL). Bilateral inferior petrosal sinus sampling (BIPSS) was done to localise ACTH secretion with stimulation by desmopressin 10 I.U. intravenously. BIPSS ruled out pituitary secretion and suggested peripheral ACTH secretion (Figure 1b). To identify the peripheral ACTH secreting tumour, contrast enhanced computed tomography (CT) of thorax and abdomen were done, which identified a 1.5 cm nodule in the apical segment of the upper lobe of the right lung with no mediastinal lymph node enlargement (Figure 1c 1d). The abdominal viscera and adrenals were normal. A core needle biopsy of the lung nodule was inconclusive on two separate attempts.

**Figure 2:**
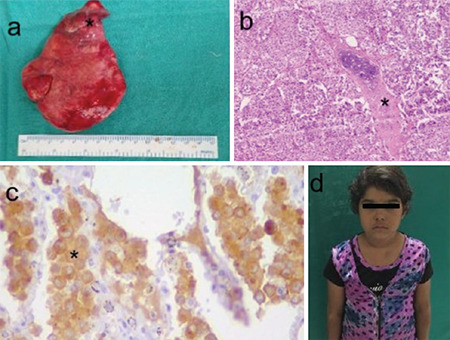
(a) Resected upper lobe of right lung, (*) tumour in apical segment, (b) haematoxylin and eosin image of nests of tumour around bronchial cartilage (*), 10x image (c) immunohistochemistry for synaptophysin, (*) showing strong cytoplasmic positivity at 40x magnification (d) postoperative photograph showing resolution of Cushing’s habitus

After management of hypokalemia and hypertension, she underwent right upper lobectomy as the location of tumour did not allow segmentectomy (Figure 2a). The histopathological examination showed a well circumcscribed tumour with a nest of small cells around bronchial cartilage (Figure 2b) and no nuclear atypia or mitotic activity. On immunohistochemistry, the cells were immune-reactive for synaptophysin and chromogranin-A (CgA) (Figure 2c) suggesting typical bronchial carcinoid.

The patient had vomiting in the immediate postoperative period for which hydrocortisone was started and tapered over a period of one week. There was persistent hypertension postoperatively for which calcium channel blocker and beta-blocker was continued. The patient became eucortisolemic three days after surgery with a cortisol concentration of 7.68 µg/dL (Table 2). Over six months of follow up, she lost 10 kg of body weight, facial puffiness and body hair have decreased and she currently does not need antihypertensives and steroids (Figure 2d). She also had normal serum calcium (9.84 mg/dL), phosphorus (6.35 mg/dL), parathormone (17.8 pg/mL) and insulin like growth factor-1 (355.7 ng/mL). Chest X-ray demonstrated expansion of the right middle and lower lobes to occupy the chest cavity.

## Discussion

### Endogenous CS

Endogenous CS is a rare disorder in children and adolescents due to increased glucocorticoids, which in early childhood is more common in males but with a female preponderance in older children (11,12). The most common cause CS is iatrogenic, due to administration of exogenous glucocorticoids (13,14). The causes of endogenous CS are classified into ACTH dependent and ACTH independent CS (Table 3).

In ACTH-dependent causes of CS, when the ACTH is produced by a pituitary adenoma, it is known as Cushing’s disease (CD).  CDis the most common cause of CS in children older than six years of age and is responsible in 75-90% of paediatric CS (11,14). Adrenal causes of CS are more common in young children and may present with features of virilisation (11,15,16). A less common cause is when ACTH is secreted by a non-pituitary tumor, known as EAS (14). EAS in young children is much rarer than in adults where it accounts for 15% of cases (8).

CS patients can present with various signs and symptoms which differ depending on patients age and causes of CS (13). Growth failure and associated weight gain are the most common presenting features of paediatric CS (13,17). Other common features are hypertension (50-60%), hirsutism (80%) and striae (61%) (17).

The differentiation between ACTH dependent and independent causes of CS is made by measuring 8 am plasma ACTH concentrations. A value of ACTH >29 pg/mL has a sensitivity of 70% to diagnose an ACTH-dependent cause of CS (11). To identify cases of CS, corticotropin-releasing hormone (CRH) test and the HDDST may be helpful but they are not reliable for differentiating between CS and other causes of ectopic ACTH secretion. This is because patients with ectopic CS may also show a decrease in cortisol level, which happened in our patient (8,13,18). BIPSS is considered to be the gold standard for lateralisation of lesions and in distinguishing CS from EAS (8,13,18). In our patient, BIPSS gradients confirmed that there was no lesion in the pituitary and thus the secretion of ACTH was ectopic.

If ACTH-independent CS is diagnosed, adrenal CT or magnetic resonance imaging (MRI) is required to differentiate between adrenocortical tumour and primary nodular hyperplasia. When an ectopic ACTH secreting lesion is suspected, CT of the neck, thorax, abdomen and pelvis should be performed to localise the lesion. Octreotide scan, positron emission tomography (PET), DOTATE scan (Ga-68 DOTA-1,4,7,10-tetraazacyclododecane-tetraacetic acid, TATE-Tyr3-octreotate) and octreotide PET scan can also help in identification of lesion (11,18). In our patient CT of the thorax using 0.5 cm sections identified a lesion of 1.5 cm in the apical segment of the upper lobe of her right lung, which was the source of the ectopic ACTH secretion.

## Ectopic ACTH Syndrome

EAS is very rare in children in comparison to adults and is more common in female children from 10 year of age (11,13). The majority of EAS cases result from carcinoid tumours of the bronchus or thymus (9) but have been reported from appendiceal, kidney and duodenal tumours (10,19). EAS may also occur due to ACTH secretion from adrenal neuroblastoma, clear cell sarcoma, pancreatic tumour, gastrinoma, pheochromocytoma, Wilm’s tumour and sacrococcygeal tumour (10,20,21,22,23,24,25). Muscle weakness, hypertension and hypokalemia is significantly more common in patients with EAS compared to those with CS (8,10). In addition, when compared to CS, patients with EAS have statistically significant higher levels of urinary free cortisol, ACTH (sensitivity 80% and specificity 74% for ACTH levels of 1.6 times the upper limit of normal) and mean ACTH increase is lower on CRH testing (sensitivity 83% and specificity 81% for differential increase of 31% in plasma ACTH) (8). Inferior petrosal sinus sampling is considered to be the gold standard for the diagnosis of EAS (10). The localization of the ACTH secreting tumor is difficult, and CT, MRI and octreotide scan, should all be used to find the tumour in EAS (10). Although biochemical tumor markers are less helpful, serum calcitonin can be used as it is known to be elevated in carcinoid tumours, medullary thyroid cancer and neuroendocrine tumors and is normal in CS (10). The surgical resection of an ACTH producing tumor is the optimal treatment, but bilateral adrenalectomy is required in refractory cases to control hypercortisolemia (10).

## Bronchial Carcinoid Tumours in Children

Although bronchial carcinoid tumours are the most common intrabronchial primary tumour in children (9,26) only 4% of them are associated with CS (27). Bronchial carcinoid tumours can arise from main, lobar or segmental bronchi and they can present with obstructive symptoms including atelectasis, dyspnea, pleuritic pain or obstructive pneumonitis (28) although our patient did not have any respiratory symptoms. Carcinoid tumors arise from Kolschitzky cells found in the basal layer of the bronchial epithelium. The overall incidence is 3-5 tumours per million people per year. However, the exact incidence of bronchial carcinoid tumours in children is not known but they constitute 70-80 percent of all primary malignant lung tumors in children (2,27,29).

Contrast-enhanced CT of the chest, with 5 mm thick sections or MRI of neck, chest and abdomen is considered first line for diagnosing ectopic ACTH secreting lesions (18,27,30). In our patient, the suspicious lesion was detected on chest CT with 5 mm sections. Octreotide scan may be useful to diagnose the primary lesion and to detect metastasis and recurrence of carcinoid tumours (9,29) but some studies suggested it to be less helpful in bronchial carcinoids, as one third of them do not express somatostatin receptors (31).

Travis et al (32,33) reported the tumour appearance as follows. Grossly, the cut surface was a homogenous tan colour with foci of haemorrhage. Microscopically they were composed of small uniform cells arranged in a mosaic pattern with interlacing fibrovascular stroma (32). The average size of these tumours is 2-4 cm and they may infiltrate the bronchial wall and surrounding lung tissue (33). The prognosis depends upon histology, lymph node status and size of tumour (30). They are classified as atypical (10%) and typical (90%) carcinoids depending upon the presence or absence of necrosis and elevated mitotic index (>2 mitoses/HPF) (9,33). Both of them can be positive for biomarkers including Chromogranin A (CgA) and synaptophysin. Typical carcinoid tumours tend to be central in location while atypical tumours tend to be peripheral (9). Although typical carcinoid tumours are considered to be benign, both variants are capable of metastasizing to regional lymph nodes, liver, bones and brain (30,33). Our patient had a typical carcinoid tumour with no evidence of necrosis and a low mitotic index and thus a good prognosis.

The treatment of choice for a bronchial carcinoid tumour is complete surgical resection with removal of involved lymph nodes (9,27). Lymph nodes are involved in up to 20 percent of paediatric cases of both types (9). Lymph node resection is more important for atypical carcinoid tumours owing to the greater malignant potential. Radiation and chemotherapy can be used where complete surgical resection is not possible (31). Somatostatin analogues, interferon α and temozolomide analogues have been used in adults with advanced disease (9). The surgery should be parenchymal-preserving whenever possible and sleeve resections and bronchoplastic procedures should be considered for central lesions (34). In our patient, it was not possible to remove the tumour while preserving the upper lobe.

A typical carcinoid tumour has a good 5-year survival rate of 88-92% and that of atypical carcinoid ranges from 60-75% (9,28,30,35). The ACTH secreting bronchial carcinoid tumours are considered aggressive variants, as lymph node positivity and recurrences are observed, even in typical carcinoid tumours (11,36). Annual serum ACTH and tumour markers should be measured as part of follow-up in order to achieve early detection of recurrence (27). CT scan of neck and chest every six to 12 months is required in node positive cases (9).

Although the surgical removal of the source of the hypercortisolaemia is the treatment of choice, medical agents such as antihypertensives and inhibitors of steroidogenesis, such as metyrapone and ketoconazole, can be used in the preoperative period to reduce the surgical risk, or when surgery is contraindicated and in postoperative period when the patient is not cured by surgical resection (18). The resolution of hypertension is more common in children compared to adults, due to vascular protective mechanism and shorter lasting hypercortisolemia (37) as is seen in our patient who became normotensive within three months after surgery.

There was requirement for postoperative hydrocortisone in our patient, as she developed postoperative vomiting, headache and weakness, which was tapered and discontinued over a period of one month. This is similar to other reports (18,38) where discontinuation of hydrocortisone within 1-2 years was noted.

The postoperative treatment of CS is challenging with a high prevalence of hypertension, increased BMI and visceral fat mass, impaired cognitive functions and decreased quality of life (18,39,40,41). However, our patient lost 10 kg weight and performing well in school. However, her growth chart needs close monitoring since patient did not have CS. Following medical or surgical treatment for CS, monitoring of growth and pubertal development is important, as growth hormone deficiency is the most common pituitary deficiency in children with CS, followed by ACTH deficiency (18).

The complete remission of CS is a rare phenomenon, which was achieved successfully in our patient by multidisciplinary approach and she has recovered completely from hypertension and Cushing’s habitus. The timely diagnosis of cause of hypercortisolism and its appropriate management is the cornerstone of successful management.

## Conclusion

Paediatric pulmonary carcinoid tumours causing EAS and leading to CS is a very rare entity and the algorithm of investigations should be followed to reach to diagnosis. BIPSS is the investigation of choice to differentiate EAS CS from CS. CT of neck and chest will help to locate the site of EAS tumours. The treatment of choice is surgical resection of tumor and involved lymph nodes with the intention of achieving negative margins and to preserve as much lung parenchyma as possible The complete remission is possible in children with EAS but bronchial carcinoid tumours causing EAS  are aggressive in nature making good follow up mandatory in these cases to monitor for recurrence.

## Figures and Tables

**Table 1:**
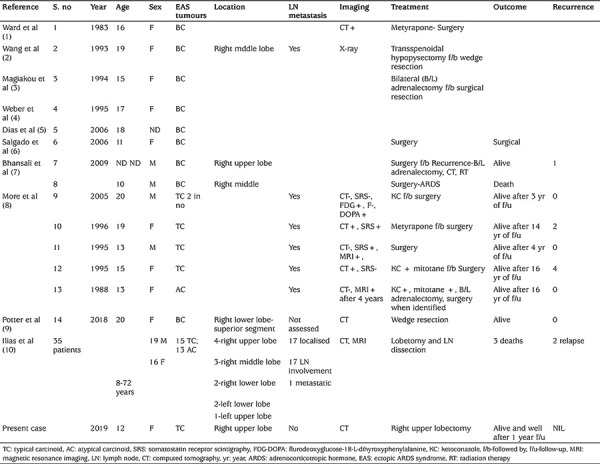
Review of literature of bronchial carcinoid tumours causing ectopic adrenocorticotropic hormone syndrome

**Table 2:**
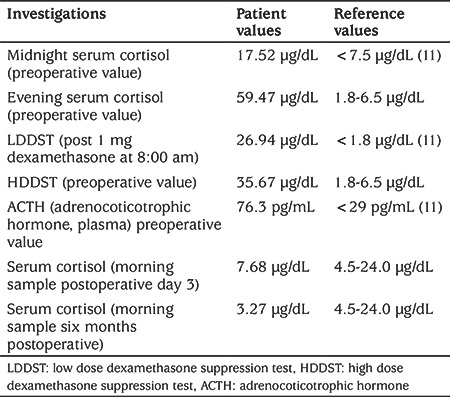
Patient cortisol and adrenocorticotropic hormone concentrations at various points during diagnosis and treatment

**Table 3:**
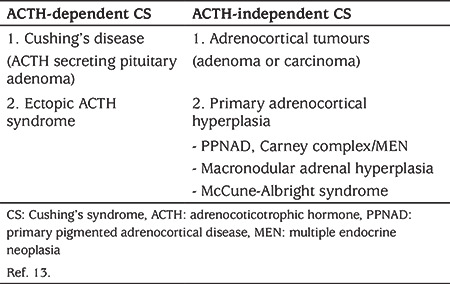
Causes of endogenous Cushing’s syndrome in children
